# Developing a predictive model for anticipating technology convergence: A transformer-based model and supervised learning approach

**DOI:** 10.1371/journal.pone.0326417

**Published:** 2025-06-26

**Authors:** Mokh Afifuddin, Wonchul Seo

**Affiliations:** 1 Textile Community College of Surakarta, Surakarta, Central Java, Indonesia; 2 Major in Industrial Data Science and Engineering, Department of Industrial and Data Engineering, Pukyong National University, Busan, South Korea; Shifa Tameer-e-Millat University, PAKISTAN

## Abstract

This study proposes a novel approach to anticipating technology convergence in the bio-healthcare sector by integrating text mining based on transformer models and supervised learning methodologies. The overarching goal is to develop a robust method for predicting technology convergence, leveraging the interrelationships between technology topics extracted from patents and research articles. Through the application of advanced techniques and by leveraging the strengths of transformer-based models such as BERTopic with KeyBERT and OpenAI integration to generate technology topics, we identified potential convergence opportunities and explored emerging trends within the dataset. The proposed method seeks to predict technology convergence effectively by employing various machine learning and deep learning techniques to train prediction models by integrating technological similarity, link prediction measures, and causal relationships between technology topics as input features, offering a more accurate and comprehensive understanding of the intricate relationships within the technological landscape. This study contributes to the literature on technology convergence by offering a novel methodology for anticipating future trends and identifying opportunities for interdisciplinary collaboration in the bio-healthcare sector. Overall, the outcomes of this study hold significant implications for businesses seeking to capitalize on emerging convergence opportunities for sustainable growth.

## Introduction

Recent decades have seen a notable acceleration of technological progress, which has resulted in the regular appearance of new technologies that provide a vast array of technical opportunities. In recent years, the phenomenon of technological convergence has drawn a lot of attention [1]. In reality, numerous sectors give rise to newly emerging industry sectors, providing prospects through technology innovation and convergence [2]. Technological convergence, a burgeoning trend in innovation across multiple sectors, involves the integration of at least two pre-existing technologies [3]. This fusion not only results in the creation of hybrid technologies but can also lead to the establishment of entirely new technological domains [4]. This convergence is the driving force behind some of the most transformative developments of this time.

Anticipating technology convergence is crucial for researchers, industry professionals, policymakers, and investors to stay ahead and seize new opportunities [5]. Understanding convergence patterns helps companies find new markets, design innovative products, and create new business models [6]. Policymakers can craft better policies to support emerging technologies. However, predicting technology convergence is complex, requiring an understanding of the relationships and evolution of different technologies. Traditional methods often fall short in capturing this dynamic process. Therefore, a more sophisticated approach is needed to identify potential convergence points among previously unrelated technologies, paving the way for innovation and progress.

Numerous models and methodologies have been developed to study technology convergence, leveraging diverse data sources such as patents [7–13], news articles [14], research articles [15], and Wikipedia hyperlinks [16]. These approaches provide critical insights into the dynamics of technology convergence across various domains. Methodologically, existing studies can be grouped into three main categories: patent citation or bibliographic [17–19], co-classification [3,9,10,12,20–22], and semantic-based methods [13,23–29]. Recently, text mining methods have become prominent for uncovering hidden relationships between technologies [13,25,26,30–36]. These techniques offer valuable insights into technology convergence, uncovering trends and relationships within the technology landscape [37,38]. Particularly, employing topics as the representation of documents has proven highly effective due to the comprehensive information they encapsulate. Technology topics, derived from the distribution of diverse keywords, offer a richer portrayal of documents when compared to other representations.

Despite the considerable value of text mining using topics, prior research predominantly relies on the conventional Latent Dirichlet Allocation (LDA) technique [39]. However, LDA may not capture complex semantic relationships. Therefore, this study seeks to improve on that by using transformer models for text mining to develop technology topics. This innovative approach harnesses the capabilities of transformer models, such as BERTopic [40], to derive richer and more contextually nuanced representations of technology topics, capturing semantic relationships better than traditional methods. Then, we will utilize two primary data sources: patents and research articles, to generate comprehensive technology topics. Next, we will use supervised learning with various machine and deep learning algorithms to train multiple prediction models. These models will leverage the connections between technology topics, incorporating measures like similarity, link prediction, and technological influence. Our goal is to create a strong method for predicting technology convergence, offering insights for R&D planning and competitive advantage. This study will provide valuable insights into emerging convergence opportunities in the evolving technology landscape.

The remainder of this paper is structured as follows. Section 2 reviews the relevant literature on technology convergence and transformer-based text mining approaches. Section 3 outlines the research methodology, including data collection, topic modeling using BERTopic, and feature extraction. Section 4 details the implementation of the supervised learning model for predicting convergence. Section 5 presents and discusses the results, with a comparison to prior studies. Finally, Section 6 concludes the paper and offers directions for future research.

## Related work

### Technology convergence

Technology convergence has been a subject of interest for many researchers and practitioners in recent years. Research on technology convergence originated in the 1960s with by Rosenberg’s recognition of overlapping technologies across industries, linking convergence to industrial progress. Agarwal and Brem [41] subsequently defined it as the integration of multiple technologies to generate innovative products, services, or systems. The literature on technology convergence spans various approaches and methodologies, emphasizing the evolution and prediction of technological trends. Research on technology convergence can be categorized into two primary streams based on their objectives: those aimed at identifying historical convergence trends [15,18,42] and those focused on forecasting future convergence patterns [8,9,12,16,43,44].

Researchers have explored diverse data sources, including patents, news articles, and Wikipedia hyperlinks, to uncover patterns and trajectories of technology convergence. Patent data is a key result of technology research, and analyzing it helps explore detailed technology trends. Technology convergence can be determined by co-occurrences of patent classification codes in a patent, such as the International Patent Classification (IPC) codes, Cooperative Patent Classification (CPC), and United States Patent Classification (USPC) [5,7,10,21,22], or by examining relationships among patent classification codes through patent citations [17,18,43,45–48]. However, predicting convergence using patent citation and co-classification methods has challenges due to ambiguity in technology classification. This ambiguity can lead to imprecise categorization, as connections between patents or classifications may not always reflect true convergence [49]. To address these challenges, various strategies have been proposed to predict the emergence of new technology convergence. Recent studies have turned to advanced techniques like text mining or semantic analysis, network analysis, and machine learning techniques to identify technology patterns [8,12,13,20,23,50,51]. Text mining methods offer a more nuanced representation of technology topics by capturing intricate semantic relationships and contextual nuances. These approaches have shown promise in revealing hidden relationships between technologies and enhancing our understanding of technology convergence dynamics [13,25,27,30,32,33,38,42,52].

### Text mining approach for technology convergence

As the landscape of innovation evolves, predicting technology convergence is crucial for strategic decision-making, innovation planning, and fostering interdisciplinary advancements. Text mining, leveraging natural language processing (NLP) techniques, has become as a valuable tool for uncovering patterns and trends in large textual datasets [53–57]. Recently, text mining has emerged as a pivotal tool for understanding and predicting technology convergence. Prior research has extensively explored text mining methodologies include LDA to uncover patterns of technological opportunities [11–13,24,42,58–60]. They have improved the performance of text-based classification and sentiment analysis tasks by utilizing advanced deep learning and word embedding techniques. They emphasized the possibility of contextual learning and semantic representation in obtaining significant patterns from unstructured texts. Additionally, by highlighting the adaptability and scalability of text mining techniques across a range of applications, some studies have combined optimization algorithms and molecular simulations to improve model accuracy and provide domain-specific insights. Kim and Sohn [23] and Feng [61] employed the document-to-vector (Doc2Vec) technique to generate vector representations for each technological domain, while Ma et al. [62] and Liu et al. [33] applied a semantic analysis using Subject-Action-Object (SAO) and topic modeling for technology convergence in emerging fields, aiming to unveil latent semantic relationships among technology topics. Afifuddin and Seo [52] demonstrated the application of semantic analysis in text mining to uncover hidden relationships between technologies over time by applying Dynamic Topic Modeling (DTM).

However, previous approaches often face limitations in capturing nuanced semantic relationships and contextual intricacies within documents. BERTopic [40] addresses this by combining the strengths of transformer-based models and advanced topic modeling techniques. Using the BERT (Bidirectional Encoder Representations from Transformers) model [63], BERTopic extracts coherent topics from textual data by capturing semantic meaning at the sentence level. Unlike traditional probabilistic models, BERTopic leverages contextual embeddings from transformers for more nuanced topic extraction. This approach has exhibited promising outcomes in topic modeling across diverse domains, as evidenced by studies conducted by An et al. [64], and Jeon et al. [65]. Therefore, this study aims to contribute to this evolving field by proposing a novel methodology that combines text mining based on a transformer model with supervised learning. By examining the interrelationships between technology topics extracted from patents and research articles, the study seeks to advance predictive modeling for technology convergence, offering a more accurate and comprehensive understanding of technological relationships. The outcomes of this research hold significant implications for businesses seeking to capitalize on emerging convergence opportunities for sustainable growth.

## Methodology

**Fig 1:**
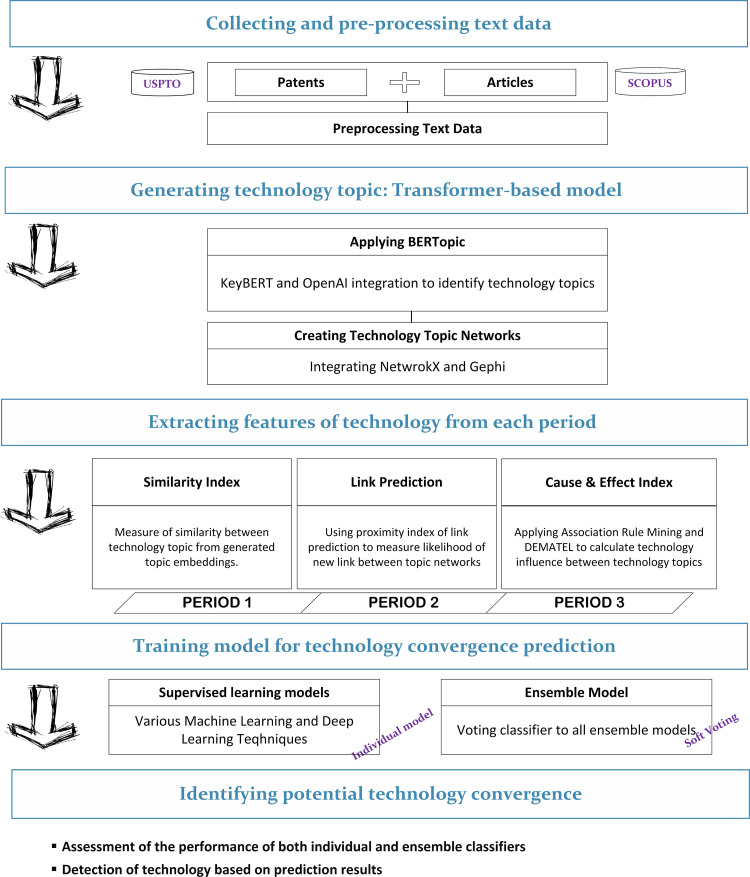
The proposed research framework for identifying technology convergence.

Illustrated in Fig 1, the comprehensive research framework delineates four key stages: (1) collecting and pre-processing text data; (2) generating technology topics with a transformer-based model; (3) Extracting features of technology from each period; (4) Training model for technology convergence prediction; (5) Identifying potential technology convergence.

### Collecting data and preprocessing

In the data collection stage of anticipating technology convergence, a crucial source involves gathering information from patent documents and research articles. Patents are valuable sources of technological advancements, offering insights into new inventions and emerging trends [10]. Research articles provide essential contextual information, explaining the scientific and technical aspects behind innovations [15]. Combining these sources enhances the predictive model’s ability to capture the dynamics of technology convergence, ensuring a strong foundation for anticipating future developments at the intersection of various technological domains [66]. In this study, we collect patent documents from the United States Patent and Trademark Office (USPTO) database, recognized for its extensive global coverage [67]. Research articles are collected from the SCOPUS database, a reputable and widely used platform for scholarly publications. We use a consistent query across both patents and research articles, focusing on the bio-healthcare domain. The collected textual data includes titles, abstracts, and claims from both patents and articles. This systematic approach ensures a comprehensive and inclusive retrieval of relevant information from both patents and articles. Table 1 shows a summary of data collection information.

**Table 1:** Overview of data gathering details.

Patent document database	https://www.wipson.com
Research paper database	https://www.scopus.com
Query formulation	“(bio AND healthcare) OR (bio-healthcare) OR (smart AND healthcare) OR (smart-healthcare) OR (digital AND healthcare) OR (digital-healthcare) OR (digital AND bio AND healthcare) OR (healthcare AND device) OR (healthcare-device)”
Publication date	2013-2021

The complexity and variability of language in patents and articles necessitate careful preparation. Initially, raw text data is subjected to a series of preprocessing techniques to standardize and enhance the quality of extracted technology topics [68]. The preprocessing begins with the removal of irrelevant characters and special symbols and the formatting of artifacts, ensuring a cleaner and more uniform text corpus.

### Generating technology topics with a transformer-based model

We used advanced methods to create technology topics from a large dataset of patents and research articles. Our approach relied on transformer models predicated on the principle of self-attention mechanisms, which excel at understanding the context of words and capturing complex relationships. Making the most of transformer-based models’ potential, specifically BERTopic [40], our approach involved encoding and clustering textual information to identify coherent and representative technology topics.

**Fig 2:**
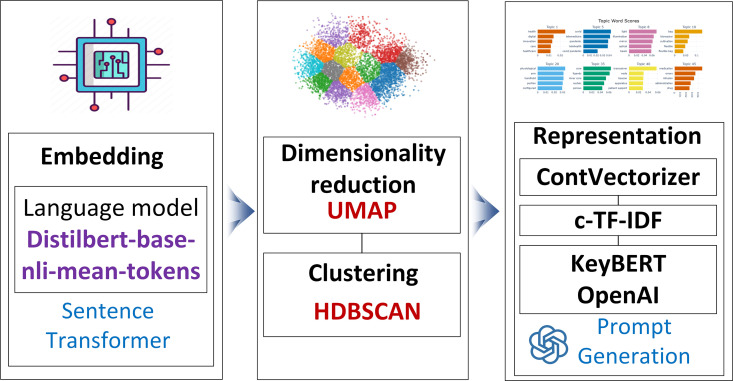
Applying the BERTopic process for generating technology topic.

The BERTopic process for generating technology topics involves several steps to extract meaningful insights from the text data, as shown in Fig 2. It starts by using BERT [63] to create embeddings, representing words in a contextualized manner. In this study, we use the DistilBERT model with the “distilbert-base-nli-mean-tokens” architecture, capturing the semantic representations of the input text. These embeddings are then reduced to a lower-dimensional space using a uniform manifold approximation and projection (UMAP) [69] while preserving word relationships. HDBSCAN [70] is then applied to cluster these embeddings into coherent groups, forming our initial technology topics. To refine these clusters, CountVectorizer is used to represent documents as numerical vectors based on term frequency. The next, Class of Term Frequency-Inverse Document Frequency (c-TF-IDF) [40], is applied to weigh the importance of terms across the entire dataset, providing a comprehensive measure of term relevance within each technology topic. Through the utilization of c-TF-IDF, BERTopic identifies key terms and their significance, constructing high-density clusters for distinct technology topics. Finally, KeyBERT and OpenAI are applied to select representative terms for each topic. This systematic approach ensures that resulting topics not only capture semantic nuances but also offer clear representations of diverse technological domains.

### Extracting features of technology topics from each period

This study develops supervised learning models to identify new opportunities for technology convergence. A crucial step is feature extraction, identifying key indicators of potential convergence between technology topics. We use three types of features: technological similarities, link prediction measures within topic networks, and causal relationships between technology topics. By analyzing these features, we aim to create a model that offers valuable insights into emerging trends and collaborative dynamics between different technological domains.

1) Measure of similarity analysis

The utilization of technological similarity between technology topics is a key aspect of our approach to understanding and predicting technological convergence. It measures how closely related different technology topics are, based on shared characteristics and functionalities. We use cosine similarity to quantify this, which compares vectors representing technology topics [71].

**Fig 3:**
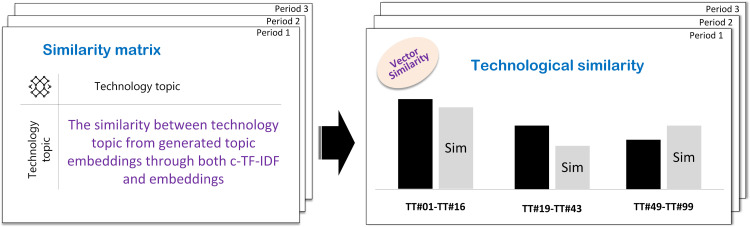
Exploration of technological similarity between technology topics.

We encode technology topics into numerical vectors using methods like word embeddings, utilizing BERTopic’s document-topic distribution. We then calculate cosine similarity between these vectors, giving us similarity scores for both documents and words. By combining these scores, we get a comprehensive representation of technological similarity. A higher cosine similarity indicates a closer relationship between topics, suggesting a higher likelihood of convergence. We have n topic vectors $T_{1},\;T_{2}\ldots .T_{n}$. The similarity matrix S is an n x n  matrix where each element $s_{ij}$ represents the cosine similarity between topics i and j. This approach helps identify potential convergence opportunities by revealing patterns and trends. The process is illustrated in Fig 3.

2) Link prediction index

**Fig 4:**
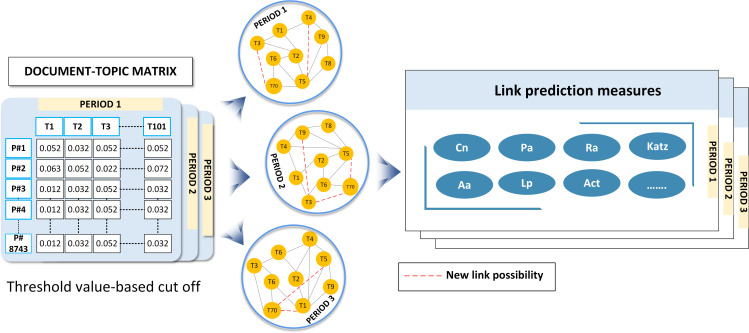
Constructing technology topic networks and applying link prediction measures to calculate proximity values for potential technology connections.

We build a co-occurrence network using the topics and apply the link prediction measures to the network to calculate proximity values for potential technology connections, as shown in Fig 4. The link prediction index is another crucial tool applied to forecast the emergence of new connections between technology topics. This index evaluates the likelihood of a link forming between nodes in the network, predicting future collaborations or convergences [72]. By assessing the historical co-occurrences and relationships, we can anticipate the evolution of connections and identify areas primed for future technological convergence.

**Fig 5:**
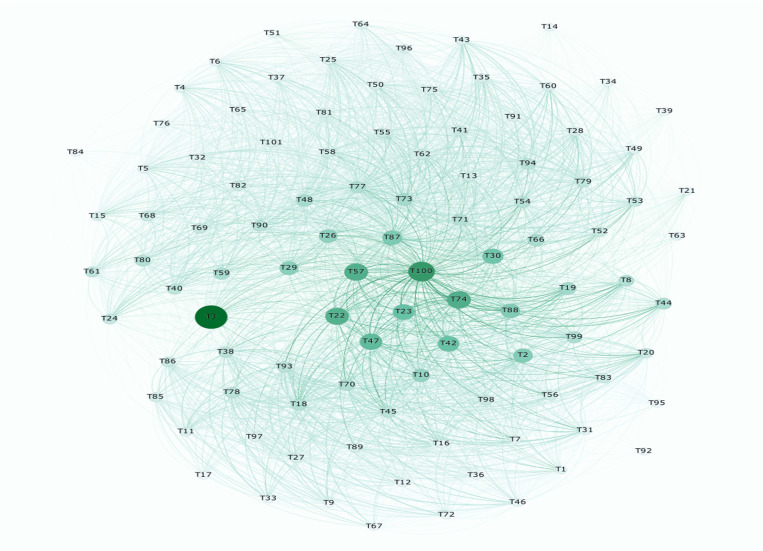
Illustration of technology topic networks in period 1(2013-2015).

Represent the technological landscape as a network where nodes represent distinct technology topics and edges signify potential connections or convergence between these topics, as shown in Fig 5. In this study, we employed a network-based link prediction approach, which includes various types of measures. This proximity index quantifies the relationships between nodes, assessing factors such as similarity, distinctiveness, and universality [44]. By integrating multiple measures, our approach aims to provide a comprehensive understanding of the dynamic interactions within the technological landscape, offering nuanced insights into the likelihood of convergence between diverse technology topics. Table 2 presents a concise summary of the proximity measures utilized in our network-based link prediction methodology, each contributing a distinct perspective to the analysis of technological relationships.

**Table 2:** The structural proximity index utilized gauges the collection of neighboring nodes and the node’s degree within a set.

Measurement	Name	Definition	Reference
Technological similarity	Jaccard Coefficient (jc)	$S(x,y)=\frac{\left|\gamma (x)\cap \gamma (y)\right|}{\left|\gamma (x)\cup \gamma (y)\right|}$	[73]
	Common Neighbor (cn)	S(x,y)=|γ(x)∩γ(y)|	[74]
	Leicht-Holme-Newman (lhn)	$S(x,y)=\frac{\left|\gamma (x)\cap \gamma (y)\right|}{k_{x}\;x\;k_{y}}$	[75]
	Hub Depressed Index (hdi)	$S(x,y)=\frac{\left|\gamma (x)\cap \gamma (y)\right|}{max(k_{x},k_{y})}$	[76]
Technological distinctiveness	Adamic-Adar (aa)	$S(x,y)=\sum_{z\in \gamma (x)\cap \gamma (y)}\frac{1}{log|\gamma (z)|}$	[77]
	Resource Allocation (ra)	$S(x,y)=\;\sum_{z\in \gamma (x)\cap \gamma (y)}\frac{1}{\left|\gamma (z)\right|}$	[78]
Technological universality	Preferential Attachment (pa)	S(x,y)=|γ(x)| x |γ(y)|	[79]
	Katz Index (katz)	$S(x,y)=\sum_{l=1}^{\infty }\beta^{l}\left|paths_{x,y}^{\left(l\right)}\right|=\sum_{l=1}^{\infty }\beta^{l}{\left(A^{l}\right)}_{x,y}$	[80]
	Average Commute Time (act)	$S(x,y)=\;\frac{1}{m(x,y)+m(y,x)}$ Where m(x,y) represents the average number of steps taken by the random walker to reach y, starting from x	[81]
Technological nearness (quasi-local)	Local Path Index (lp)	$S=A^{2\;}+\;\in A^{3}$ Where A denotes an adjacency matrix for nodes x and y, while ∈ is a free parameter	[78]

3) Linkages of technological influences

Determining technological influence is a multi-step process involving rule mining, rule mining measures, and the application of the DEMATEL for a comprehensive analysis that considers both direct and indirect effects [82]. First, rule mining extracts meaningful patterns and relationships between technology topics based on their connections or co-occurrences. This helps identify associations and interactions within the technological landscape. Next, rule mining measures quantify the strength and significance of these extracted rules by assessing frequency, confidence, and support, providing a quantitative basis for evaluating relationships between topics. Then, the DEMATEL technique is applied to analyze the influential effects of technology topics that consider both direct and indirect effects. The formula of the DEMATEL method involves the following:

- Construction the direct-relation matrix (D):

$$D=\;\left[\begin{array}{c} \begin{array}{ccc} d_{11} & d_{12} & \ldots \;d_{1n}\\ d_{21} & d_{22} & \ldots \;d_{2n}\\ \vdots & \vdots & \ddots \;\vdots \end{array}\\ \begin{array}{ccc} d_{n1} & d_{n2} & \ldots \;d_{nn} \end{array} \end{array}\right]$$ (1)

- Normalize the direct-relation matrix

$$N=\;\frac{D}{Max\;(\sum_{i=1}^{n}d_{ij},\;\sum_{j}^{n}d_{ij})}$$ (2)

- Calculate the total-relation matrix

$$T=\;{N\;(I-N)}^{-1}\;$$ (3)

Where I is the identify matrix of the same size as N

- Determine the influential degree and relationship

$$r_{i}=\;\sum_{j=1}^{n}t_{ij}\;(Total\;influence\;extracted\;by\;factor\;i)$$ (4)

$$c_{j}=\;\sum_{i=1}^{n}t_{ij}\;(Total\;influence\;received\;by\;factor\;j)$$ (5)

**Fig 6:**
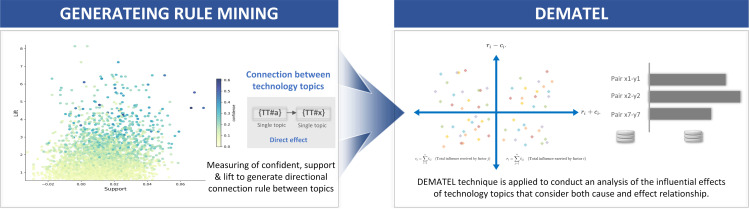
Exploration of cause-and-effect relatedness between technology topics.

The result is a thorough and data-driven exploration of technological influence, aiding in the identification of key players and themes that shape the dynamics of technological convergence. Fig 6 illustrates the investigation of causal relationships between technology topics.

These feature extraction processes contribute to the development of predictive models capable of navigating and predicting the evolving landscape of technology convergence, offering insights into emerging trends and collaborative opportunities.

### Training classification model for technology convergence prediction

In training the classification model for predicting technology convergence, input features for our model encompass key metrics such as cosine similarity, link prediction index, and technological influence measures. The input features used in exploring technology convergence in this study are detailed in Table 3. Following the extraction of these features during period 1 and the observation of technology convergence in period 2, we applied them to train various prediction models. Due to the inherent variability in the ranges of values among these features, normalization is imperative during the model training process.

**Table 3:** Input features employed for training models to analyze technology convergence.

Type	Feature	Description
Similarity measure	Index_cs	Cosine similarity between technology topic from generated topic embeddings.
Link prediction	Index_cn Index_jc Index_pa Index_aa Index_ra Index_hdi Index_katz Index_lhn Index_ac Index_lp	The likelihood of the pair of technology topics being linked in the next period is determined by a particular link prediction algorithm (such as common neighbor (cn), jaccard (jc), preferential attachment (pa), adamic-adar (aa), resource allocation (ra), hub depressed index (hdi), katz, leicht-holme-newman (lhn), average commute (ac), and local path index (lp).
Technological Influences	Index_cause Index_effect	The degree to which each technology topic influences all other topics. The level of influence that each technology topic receives from all others.

The model is trained using various machine learning or deep learning approaches, including Logistic Regression (LR), Support Vector Machine (SVM), Random Forest (RF), Naïve Bayes (NB), Gradient Boasting (GB), XGBoost (XGB), LightGBM (LGBM), and Deep Neural Network (DNN). Algorithms are selected to effectively capture patterns and relationships within the multidimensional input feature space. The training process involves optimizing the model parameters to enhance its predictive capabilities. To ensure the robustness and generalizability of the model, rigorous validation and evaluation procedures are implemented. The model is tested on a separate dataset to assess its performance in predicting future technological convergence and identifying influential topics. Evaluation metrics such as precision, recall, and F1 score provide quantitative measures of the model’s accuracy. Given the highly formalized nature of the input features, we are confident that employing fundamental machine learning and deep learning techniques will be adept at navigating the complexities associated with technology convergence. The model aims to provide actionable insights for navigating the ever-evolving landscape of technological convergence.

### Identifying potential technology convergence

In this study, we aim to predict future technology convergence beyond period 3. We evaluate the performance of different machine learning models using data from period 2 to predict whether convergence occurred in period 3. We assess the models using metrics like precision, recall, F1 score, and accuracy to see how well they predict convergence. By comparing these models, we gain insights into their effectiveness. Then, we analyze the predicted convergence instances to understand future convergence patterns and identify emerging trends. This analysis helps decision-making and strategic planning in various industries.

## Result and analysis

### Data exploration

**Fig 7:**
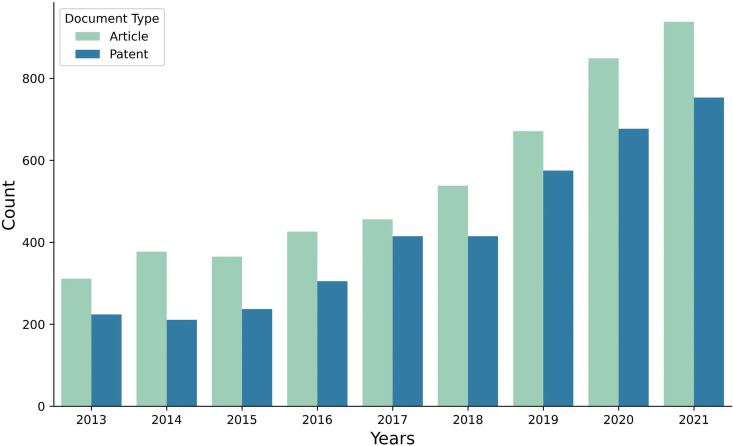
The distribution pattern of articles and patents each year in bio-healthcare field published from 2013 to 2021.

The data were collected in the bio-healthcare field from January 2013 to December 2021 and include patent documents sourced from the USPTO and research articles from SCOPUS publications. The number of patents is 3,812, and the number of research articles is 4,931. In total, 8,743 documents were carefully collected and formed the basis of our dataset for analysis and exploration. The distribution pattern of documents based on type each year is shown in Fig 7. We combine all documents and divide them into three periods based on time intervals, and each period is analyzed separately to observe technology topics within the evolving technological landscape. The distribution pattern of documents throughout the various years is seen in Table 4. The first period, 2013–2015, had 1,725 documents; the second period had 2,555 documents; and the third period had 4,463 documents.

**Table 4:** The number of all documents based on time interval.

Period	Grant Year	Number of documents
Period 1	2013	535
	2014	588
	2015	602
Period 2	2016	731
	2017	871
	2018	953
Period 3	2019	1,246
	2020	1,526
	2021	1,691
**Total**		**8,743**

Cleaning the text data includes tasks such as removing special characters, punctuation, and irrelevant symbols to ensure a consistent and standardized format. Additionally, common text preprocessing techniques like lowercasing and stemming may be applied to enhance uniformity. Once the text is cleaned, the extraction phase involves identifying and isolating key information.

**Fig 8:**
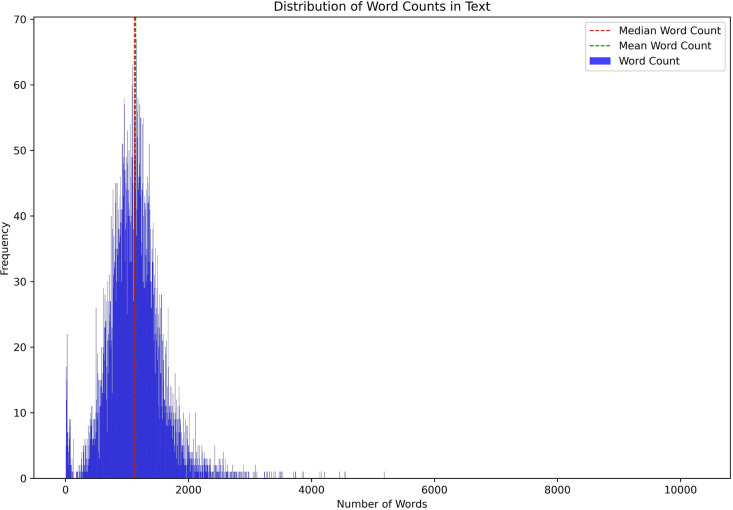
Word count distribution in the text data after preprocessing, showing the frequency of words across the dataset.

In our study, we assumed textual data could effectively represent the technology landscape. Topic modeling generates topics based on word distribution in these texts. Fig 8 shows word count distribution: blue bars indicate document counts within each range, while red and green dashed lines represent the median and mean word counts, respectively. This highlights the importance of preprocessing to extract meaningful insights from the technological landscape.

### Analysis of topic extraction and interpretation for generating technology topic

After the exploration and preprocessing of the text data, the BERTopic process is initiated to uncover latent topics within the technological landscape. BERTopic, a topic modeling technique based on transformer architecture, is applied to generate clusters of words that represent distinct technology topics. The resulting document-topic distribution reveals the likelihood or proportion of each document belonging to these identified topics. This distribution is then analyzed to extract meaningful insights into the prevalent themes and patterns within the dataset. By delving into the output of BERTopic, we gain a comprehensive understanding of how technology topics are interrelated and how they manifest in the corpus of text data.

**Fig 9:**
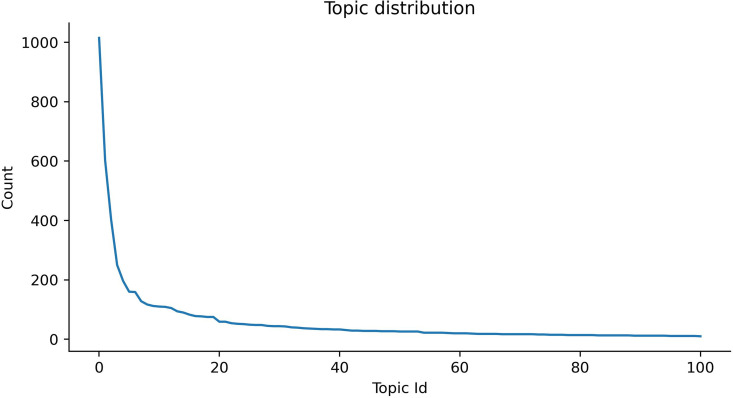
Distribution of document counts across various topics, excluding outliers.

**Fig 10:**
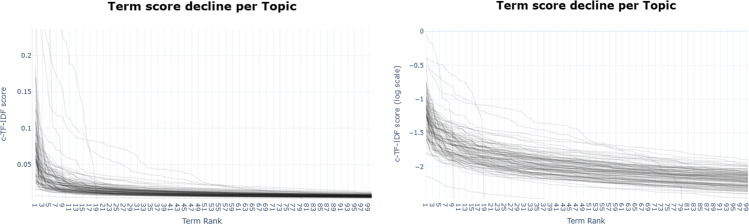
The distribution of c-TF-IDF scores across terms within topics (a) the visualization of term scores for each topic (b) the visualization of term scores with logarithmic scaling.

The BERTopic model provides information about the frequency and characteristics of each identified topic. Fig 9 illustrates the distribution of document counts across different topics, excluding any potential outlier topics. In this study, we provide a comprehensive analysis of term scores. As illustrated in Fig 10, the illustration reveals insightful patterns in the distribution of c-TF-IDF scores across terms within topics. Across the majority of topics, there is a notable decline in c-TF-IDF scores from the top-ranked term to the third-ranked term. Beyond the third rank, the scores exhibit a gradual flattening, indicating a diminishing rate of decrease as the rank increases.

**Fig 11:**
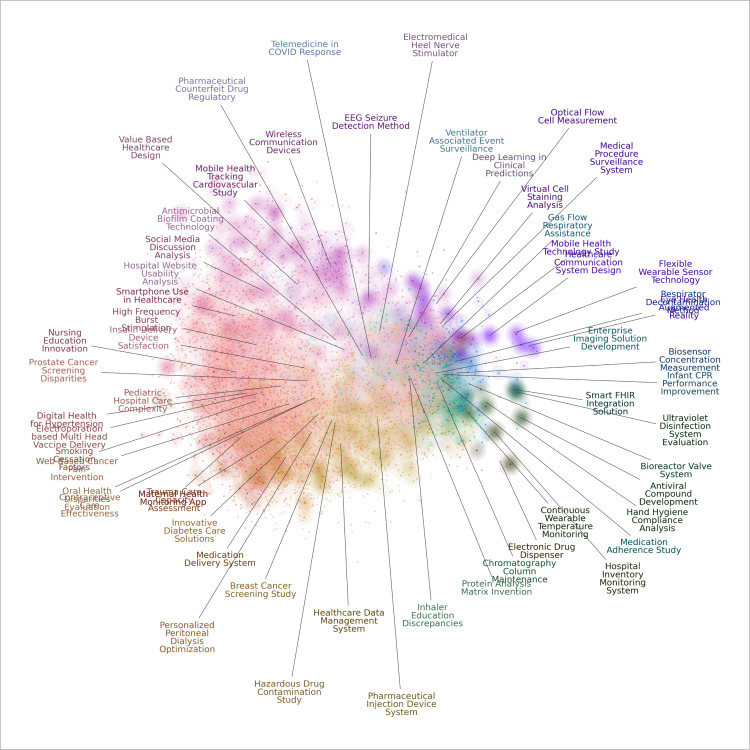
Visualization of embedding reduction with fine-tune topic representation.

Finally, we use KeyBERT and OpenAI to choose representative terms for each topic and to generate well-crafted labels for our topics. By integrating prompt generation with OpenAI, BERTopic not only clusters related documents into meaningful topics but also assigns them polished, informative labels. Additionally, to enhance the accessibility and comprehension of the identified technology topics, we leverage visualizations derived from the BERTopic output. Fig 11 shows a plot to visualize the embeddings generated by BERT after dimensionality reduction, with each data point labeled according to its corresponding category. This visualization helps us understand the main themes of each technology topic.

### Result of prediction model

**Fig 12:**
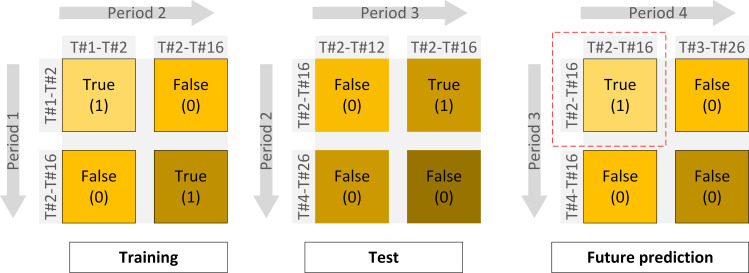
Training on labeled data in pairs to forecast whether the pairs will occur in the subsequent period.

By utilizing insights obtained from advanced features extracted, including metrics like similarity, link prediction, and technological influence, our objective is to construct a model that can offer valuable insights into emerging trends and collaborative interactions among diverse technological domains. The features derived from the identified topics will serve as inputs for a machine learning algorithm, specifically a classification model. Training on labeled data is shown in Fig 12, which consists of technology topics either connected or not connected in the subsequent period, the classification model aims to discern patterns indicative of technology convergence.

We have created eight individual classification models, each optimized with specific hyperparameters tailored for precise predictions of new technology convergence. By utilizing data from period 1, the models are trained to anticipate technological convergence in the following period. The effectiveness of the trained classification model will be assessed using a test dataset comprising pairs of technology topics where convergence was not found in period 2. The model will predict the occurrence of convergence in period 3 for these pairs. The details of the number of datasets used for training and testing are shown in Table 5. Notably, the dataset faces a class imbalance challenge, as there are fewer pairs of converged topics in the current period compared to the number of topic pairs that did not converge. Therefore, we utilize the Synthetic Minority Over-sampling Technique (SMOTE), a method commonly applied to create a balanced dataset by oversampling data samples through statistical distribution adjustments [83]. Table 6 shows descriptive statistics for input features, providing a comprehensive summary of the data’s central tendencies and overall distribution. The relative importance of each feature used in the predictive model is further detailed in Supporting information S1 Fig, highlighting their individual contributions to forecasting technology convergence.

**Table 5:** The number of pairs between technology topics to be used as training and test datasets.

Pairs not linked in period 1 (Training input feature)	Pairs linked in period 2 (Y = True)	Pairs not linked in period 2 (Y = False)
4,128	3,966	162
Pairs not linked in period 2 (Test input feature)	Pairs linked in period 3 (Y = True)	Pairs not linked in period 3 (Y = False)
2,016	1,953	63

**Table 6:** Descriptive statistics data for input features in period 1.

Index	Min.	Std.	Q1	Mean	Median	Q3	Max
cn	0.0000	12.6545	12.0000	21.4006	22.0000	31.0000	53.0000
jc	0.0000	0.1520	0.1240	0.2398	0.2323	0.3367	0.8036
pa	864.0000	807.7528	2520.0000	3096.7790	3080.0000	3678.7500	5229.0000
aa	0.0000	3.0163	2.7474	5.0514	5.1097	7.2501	12.6199
ra	0.0000	0.1932	0.1616	0.3117	0.3089	0.4525	0.8170
hdi	123.8333	7.8045	150.3333	155.4796	156.1667	161.1667	173.0000
lhn	0.0000	0.0122	0.0000	0.0108	0.0088	0.0182	0.0909
katz	−0.4659	0.1192	−0.1178	−0.0375	−0.0302	0.0517	0.2855
ac	0.1639	0.0143	0.1888	0.1982	0.1967	0.2032	0.2651
lp	0.0100	0.3528	0.5700	0.8433	0.8400	1.0400	2.1800
cs	0.0037	0.0048	0.0128	0.0163	0.0165	0.0194	0.0314
cause	0.0000	0.0008	0.0001	0.0006	0.0003	0.0008	0.0061
effect	0.0000	0.0012	0.0002	0.0010	0.0005	0.0012	0.0088

**Fig 13:**
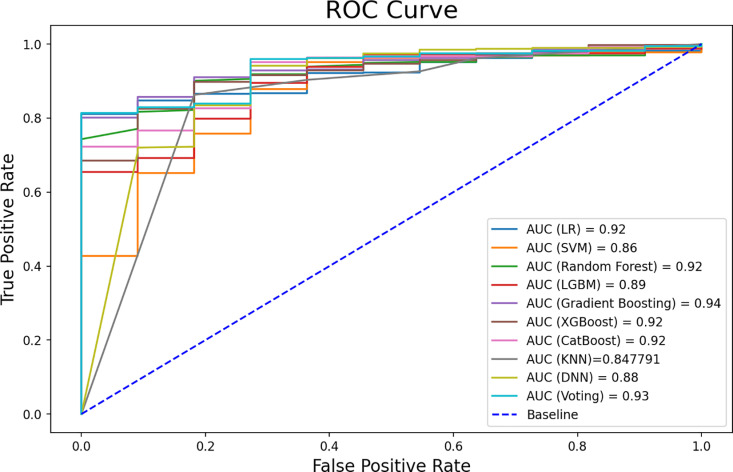
ROC curve for individual models and voting classifier.

Comparisons between the evaluation results and the actual convergence in the third period will be conducted to gauge the accuracy of the model. The insights garnered from the evaluation results, as delineated in Table 7 and Fig 13, offer valuable perspectives on the performance of the classification models. This analysis forms a critical aspect of understanding the model’s effectiveness in predicting convergence opportunities between technology topics. The table presents the performance measures based on the test data for both the nine individual models and the voting classifier. Unmistakably, it illustrates that each algorithm demonstrates diverse levels of accuracy, precision, recall, and F1 score. This comprehensive examination contributes to a nuanced understanding of the strengths and weaknesses of the classification models and the overall predictive capabilities of the voting classifier.

**Table 7:** The results from the performance of individual classification model and voting classifier.

Algorithm	Accuracy	Precision	Recall	F1 score	AUC
LR	0.779	1.000	0.773	0.872	0.933
SVM	0.863	0.988	0.870	0.925	0.891
RF	0.935	0.979	0.954	0.966	0.929
GB	0.873	0.994	0.875	0.930	0.923
XGB	0.945	0.984	0.959	0.971	0.899
LGBM	0.940	0.986	0.951	0.968	0.921
CB	0.943	0.984	0.956	0.970	0.915
KNN	0.905	0.991	0.910	0.949	0.813
DNN	0.975	0.977	0.997	0.987	0.939
Voting	0.950	0.989	0.959	0.974	0.938

The majority of models demonstrate excellent performance, with high accuracy, precision, recall, and F1 scores. The voting classifier consistently performs well across all metrics, showcasing its robustness and effectiveness in combining the strengths of individual models. The highest AUC values are observed in the voting classifier, DNN, RF, and LR models, indicating strong overall predictive capabilities. The choice of the best model may depend on the specific requirements of the application. However, the DNN stands out as an optimal choice due to its well-rounded performance across multiple metrics.

## Discussion

The objective of this study was to predict future technology convergence by analyzing patent and article data from period 3 using supervised learning. This approach aligns with the rapid pace of technological advancements. In the scope of our investigation, we specifically focused on forecasting technology convergence trends during period 4. This timeframe was selected to encapsulate significant trends and patterns in the evolution of technology topics, ensuring the relevance and timeliness of our predictions. The findings indicated that among the 101 labeled technology topics, certain pairs exhibited DNN probabilities equal to or exceeding 0.5. These high-probability pairs, highlighted in Table 8, demonstrate notable associations, suggesting promising areas of convergence. Among these pairs that have a high probability are: T12 is related to augmented reality ophthalmic displays that integrate augmented reality (AR) technology into ophthalmic displays. T4 is cardiac health, which focuses on monitoring and treating conditions in the heart. T36 is focused on holographic cell imaging, which utilizes holographic microscopy techniques to capture high-resolution images of cells and specimens. T9 is associated with sensor technology and monitoring, while T6 pertains to related orthopedic surgery and recovery.

**Table 8:** Potential technology convergence.

Pair of technology topics				Technology implication
Topic	Description	Topic	Description	
T36	Holographic Cell Imaging	T6	Orthopedic Surgery & Recovery	The combination of holographic cell imaging, sensor technology, and orthopedic surgery and recovery could greatly boost patient involvement and confidence during rehab. By using holographic visuals and sensor feedback in tailored rehab plans, patients can actively track their progress and goals, which can enhance motivation and exercise adherence. This increased engagement may speed up recovery and improve overall function. Additionally, providing real-time feedback and virtual guidance through tele-rehab platforms can widen access to care, making it easier for patients to stay on track even outside the doctor’s office.
T9	Sensor Technology and Monitoring	T6	Orthopedic Surgery & Recovery	
T12	Augmented Reality Ophthalmic Display	T4	Cardiac Health & Intervention	Combining these technologies could bring new solutions to enhance heart care, diagnostics, and treatments. “Real-time cardiac visualization with AR ophthalmic display” means creating special AR displays for heart surgeons and cardiologists. These displays can show detailed views of the heart, like its chambers and blood vessels, in real-time. They can also overlay heart imaging data onto the patient’s anatomy during surgery planning.
T89	Liquid Sample Analysis Technology	T39	Hearing Aid Solutions	The convergence of liquid sample analysis technology with hearing aid solutions has the potential to transform traditional hearing aids into multifunctional health monitoring devices that not only improve hearing but also provide valuable insights into the wearer’s overall health status. This technology could enhance early disease detection, promote proactive healthcare management, and empower individuals to take control of their well-being.
T12	Augmented Reality Ophthalmic Display	T65	Smoking Cessation Technology	The convergence of augmented reality ophthalmic display with smoking cessation technology has the potential to revolutionize smoking cessation efforts by providing personalized support, real-time health visualization, interactive resources, and virtual coaching. This technology could empower individuals to quit smoking, improve their overall health, and reduce the burden of tobacco-related diseases on society.

The opportunity for technology convergence between T12, which is related to AR ophthalmic display, and T51, which is related to breast cancer screening, presents a compelling future technological opportunity in the realm of medical imaging and diagnostics. This convergence can lead to “AR-Assisted Breast Cancer Screening” [84]. Future advancements may involve integrating augmented reality technology into breast cancer screening processes, allowing radiologists and healthcare professionals to visualize and interact with mammographic images in real-time using AR headsets or display devices. AR overlays could provide enhanced visualization of breast tissue structures, lesions, and abnormalities detected during screening mammograms, enabling more accurate interpretation and diagnosis. AR-guided tools and features could facilitate interactive analysis of mammographic images, allowing radiologists to annotate findings, measure tumor dimensions, and navigate through 3D reconstructions of breast anatomy for comprehensive assessment. The convergence of augmented reality ophthalmic display with breast cancer screening technology holds the potential to revolutionize the way breast cancer is screened, diagnosed, and managed, offering opportunities for improved accuracy, efficiency, and patient-centered care in breast healthcare.

Combining T36 (holographic cell imaging), T9 (sensor technology), and T6 (orthopedic surgery) can advance personalized orthopedic care. Develop wearable sensors (T9) to monitor patients’ movement, joint mobility, and physiological parameters before, during, and after orthopedic surgeries and rehabilitation (T6). These sensors can provide real-time feedback on range of motion, muscle strength, gait analysis, and vital signs to patients and healthcare providers. Use holographic cell imaging (T36) to create detailed 3D models of musculoskeletal structures, aiding surgeons in visualizing patient-specific anatomy for precise surgical planning. Implement AI-driven algorithms to analyze sensor data and holographic images, creating personalized rehabilitation protocols tailored to each patient’s needs and progress. Provide real-time feedback during rehabilitation with holographic overlays to help patients perform exercises correctly and avoid injury.

## Conclusion

Technology convergence involves creating new technologies by combining innovations from different fields. Anticipating this convergence is vital for driving innovation and gaining a competitive edge. This study presents a method for predicting technology convergence in the bio-healthcare sector. By combining text mining based on transformer models and supervised learning, we analyze patents and research articles to find convergence opportunities and future trends. Previous methods often missed the nuanced relationships in documents, limiting their insights into technology convergence. Our approach uses advanced techniques, like BERTopic for topic modeling, to identify potential convergence opportunities and emerging trends. We integrate technological similarity, link prediction, and causal relationships between technology topics to train machine learning and deep learning models. A voting classifier combines these models, improving performance over previous methods. This approach enhances our understanding of technology convergence, advancing predictive modeling in technological innovation.

Our analysis revealed promising convergence prospects across various technology topics, including augmented reality in ophthalmic displays, holographic cell imaging, sensor technology, and cardiac health. These findings highlight how multidisciplinary collaboration and technology integration can transform future healthcare innovation. Our results demonstrate that the transformer-based model effectively identifies nuanced and semantically rich technology topics from both patent and article data. Compared to earlier studies that relied on traditional topic modeling methods such as Latent Dirichlet Allocation (LDA) [45] or semantic analysis methods like SAO [62], our approach yielded more contextually coherent topic clusters and higher prediction performance in supervised learning. For instance, while Kim and Sohn [23] used Doc2Vec to predict convergence and improved accuracy through vector combination with bibliometric indicators, our model surpassed this by integrating topic networks and link prediction features derived directly from transformer embeddings. Additionally, compared to Giordano et al. [38], which used text and dynamic network analysis to measure convergence in defense patents, our method emphasizes topic-level convergence prediction and includes both patent and research article data for broader contextual understanding. These enhancements underscore the potential of transformer-based models in capturing the evolving and interconnected nature of emerging technologies. This comprehensive strategy not only refines predictive capabilities but also provides a nuanced understanding of relationships within the technological landscape. The research is expected to help organizations with R&D planning by providing them the ability to seize new opportunities from the convergence of specific technology topics and secure strategic competitive advantages for sustainable growth.

However, it is important to recognize the limitations of our study, including the reliance on historical data and the potential biases inherent in predictive modeling techniques. Furthermore, it is difficult to predict future convergence patterns with accuracy due to the dynamic nature of technology growth. To improve the precision and dependability of convergence forecasts, future studies in this field could explore advanced machine learning approaches and integrate real-time data sources. Studies that follow the development of technology topics over time could also provide valuable insights into the dynamics of convergence trends and their implications for innovation and industry competitiveness.

Overall, our study contributes to the growing body of literature on technology convergence by offering a novel methodology for anticipating future trends and identifying opportunities for interdisciplinary collaboration in the bio-healthcare sector. By addressing the challenges and limitations outlined in this study, we can continue to advance our understanding of technological convergence and drive transformative innovations in healthcare and beyond.

## Supporting information

S1 Fig Feature importance. (TIF)

S1 Data Feature values for model training. (XLSX)

S2 Data Feature values for model testing. (XLSX)

S3 Data Feature values for future predictions. (XLSX)
