# Supplementary figures and images for: Developing a predictive model for anticipating technology convergence: A transformer-based model and supervised learning approach
Source: PLoS One. 2025 Jun 26;20(6):e0326417. doi: 10.1371/journal.pone.0326417 (PMC12200714; doi:10.1371/journal.pone.0326417)

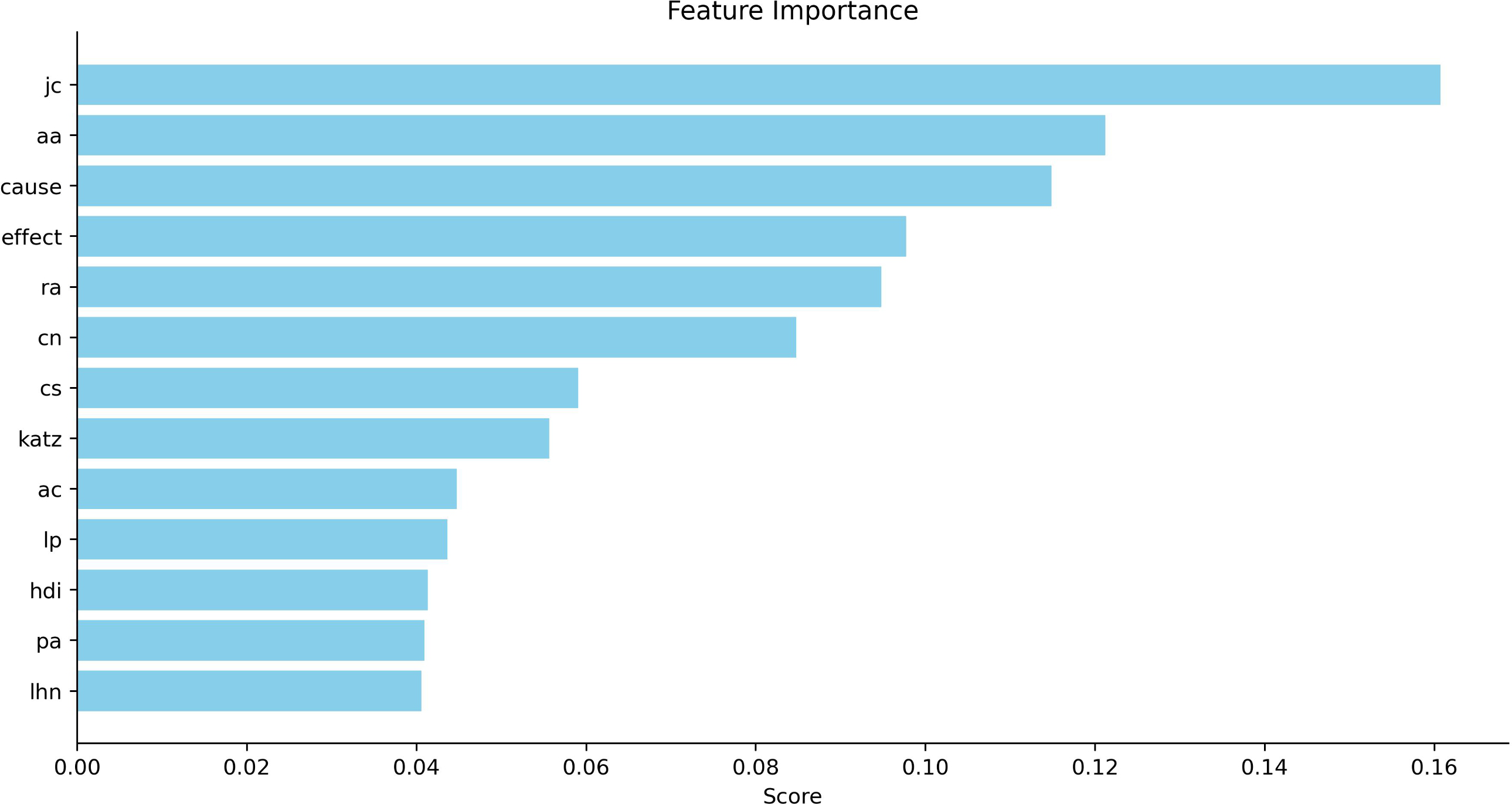

Supplement: S1 Fig — (TIF) [file pone.0326417.s001.tif]
